# Molecular polymorphisms that underlie trait variation in crops: Lessons learned from soybean

**DOI:** 10.1002/tpg2.70173

**Published:** 2025-12-30

**Authors:** Mary Jane C. Espina, Aaron J. Lorenz, Robert M. Stupar

**Affiliations:** ^1^ Department of Agronomy and Plant Genetics University of Minnesota Saint Paul Minnesota USA

## Abstract

Genetic variation within a germplasm is important in crop improvement, providing a foundation for breeders to develop new varieties. Traits of agronomic and economic importance are often mapped to identify the genetic basis of observed phenotypes, oftentimes using quantitative trait locus (QTL) analysis. While some mapped QTLs can be directly utilized in breeding programs, others benefit from a deeper understanding of the causal genes and sequence polymorphisms for effective deployment. In this review, we examine the factors that differentiate QTL where causal DNA sequence polymorphisms can be readily identified from those where causation remains difficult to resolve. We present a case study for soybean (*Glycine max* (L.) Merr.), in which we surveyed the literature to find the sequence variants underlying QTL in this crop. We cataloged causal variants by type, including variations within coding and regulatory regions, transposable element insertions and deletions, epigenetic modifications, and gene content variants (e.g., copy number variants). Additionally, we discuss the impediments to gene discovery, including challenges in phenotyping, the nature of sequence polymorphisms, and the difficulty of functional validation. Finally, we highlight future opportunities in the field of gene discovery, emphasizing how advances in high‐resolution mapping, near‐gapless genome assemblies, pangenome resources, and genome engineering will help overcome existing barriers and accelerate the discovery of causal genes and variants underlying complex traits.

## Core Ideas

- Specific circumstances determine the likelihood of identifying the causal molecular variant underlying a phenotype.
- Advances in omics technologies have aided the identification of complex causal variants.
- Variants underlying crop domestication and agricultural traits may be more likely to be caused by complex variants.

## Plain Language Summary

The identification of molecular variants that cause phenotypic variation in crops is valuable, as this opens the possibility of targeting these genes to develop new traits. These causal variants consist of a wide range of molecular variants that typically affect the coding sequence or expression of a specific gene or genes. While some causal variants are relatively easy to identify, those impacting domestication or agriculturally relevant traits are oftentimes complex and more difficult to identify. The development of new genomics and multi‐omics resources in crop research communities is making the identification of these complex variants more accessible. This paper uses soybean as a case study, describing how researchers are overcoming the obstacles of gene discovery for important traits.

**Abbreviations**
Cas9: CRISPR‐associated protein 9
CHS: chalcone synthase
CNV: copy number variation
CRISPR: Clustered Regularly Interspaced Short Palindromic Repeats
indel: insertion/deletion
lncRG: long non‐coding RNA genes
MG: maturity group
PAV: presence/absence variation
QTL: quantitative trait locus
RNAi: RNA interference
SCN: soybean cyst nematode
SNP: single nucleotide polymorphism
TE: transposable element
TILLING: Targeting Induced Local Lesions in Genomes

## 1 INTRODUCTION

Phenotypic and genotypic variation within germplasm collections is essential for crop improvement. In most breeding programs, important traits are identified and mined from these collections to find natural variations. Once identified, traits of interest are genetically mapped to associate the phenotype to underlying genomic regions. A quantitative trait locus (QTL) is a segment of the genome that contains a gene or genes that influence variation for a trait(s). Researchers can identify the positions of QTL within the genome by detecting co‐segregation between a genetic marker and a trait of interest based on genetic linkage (T. Mackay et al., 2009; Miles & Wayne, 2008). It is generally assumed that the DNA within a QTL region carries a specific causal variant, such as DNA sequence polymorphism or an epigenetic modification, that dictates the observed phenotypic variation. The causal variant for a given QTL may affect the structure or function of a specific gene product (e.g., an encoded protein per se) or influence gene regulation at the transcriptional or post‐transcriptional level (e.g., RNA interference [RNAi] mechanisms).

The QTL associated with traits of interest can be identified via coarse mapping, which can be performed using family‐based populations (bi‐parental or multi‐parent) and/or a natural‐based population via association mapping (Jaganathan et al., 2020; Y. Xu et al., 2017). Initial QTL mapping can be highly accurate (Price, 2006), and some of the major‐effect QTLs for agricultural traits have been successfully exploited by breeders for crop improvement and varietal development (J. Kumar et al., 2017). Although QTL mapping is not a prerequisite for leveraging natural variation for crop improvement, breeders exploit the identified QTL to develop molecular markers for selecting economically important traits (Bernardo, 2008). For highly polygenic traits such as yield, the use of genomewide markers or genomic selection is more appropriate than characterizing individual QTLs of small effects using some statistical methods (Heslot et al., 2015). Conversely, for traits controlled by large‐effect loci, QTL mapping serves to gain a deeper understanding of the genetic architecture of the trait of interest. Furthermore, major QTLs explaining more than 10% of phenotypic variation can be incorporated into the genomic selection model as fixed effects (Bernardo, 2014).

**FIGURE 1:**
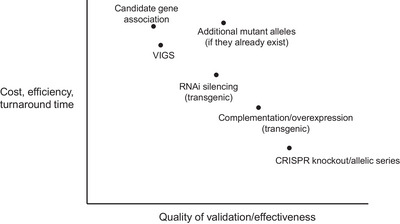
Methods of gene validation. A scatter plot illustrating various gene validation methods, their relative effectiveness, and the time it takes to validate a certain trait of interest. Note that these are subjective approximations; the cost, efficiency, turnaround time, and effectiveness of validation methods vary among traits and experimental circumstances. Also note that “Candidate gene association” alone does not constitute a full functional validation. CRISPR, Clustered Regularly Interspaced Short Palindromic Repeats; RNAi, RNA interference; VIGS, virus induced gene silencing.

**FIGURE 2:**
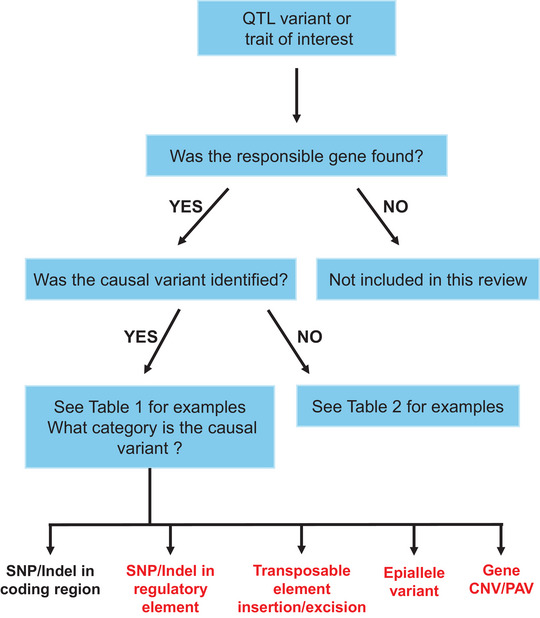
Definitions of gene discovery. Flowchart outlining the criteria used to determine whether a gene and its associated causal variant were identified for a quantitative trait locus (QTL) or trait of interest. The red text indicates categories considered “complex variants.” This review focuses on natural variants; induced genetic variants are not included in the Table 1 analysis nor discussed in detail.

QTLs of great biological, economic, and/or agricultural significance may become the subject of fine‐mapping, which involves the use of high‐resolution markers to narrow the chromosomal interval, ultimately identifying a smaller region containing a list of candidate genes for further investigation (Salvi & Tuberosa, 2005). Within the refined interval, candidate genes are typically subjected to positional cloning to pinpoint the location of the causal gene and polymorphism. This can be achieved, for example, by developing near‐isogenic lines to screen a large number of progenies in a uniform genetic background and finding more recombination events within a small genetic interval (Remington et al., 2001). Positional cloning relies on the relationship between recombination and physical distance, where recombination events resolve the co‐segregation pattern of markers and phenotypes in fine detail, thus reducing the physical genetic space attributed to the QTL (Bortiri et al., 2006). Once an approximate position has been established, candidate genes within the region can be prioritized by examining functional annotations, homology to known genes, gene expression profiles, sequence polymorphisms, and involvement in known biological pathways (Tranchevent et al., 2011). Selected genes are then subjected to candidate gene validation experiments to confirm their involvement in the observed phenotype of interest (Pflieger et al., 2001). Figure 1 shows the range of functional validation approaches available to researchers, positioned approximately according to their accessibility and reliability for validation. A gene that is functionally validated by one or more of these methods is said to be identified as the gene responsible for a given trait or QTL. Some genes can be identified without a full understanding of the sequence polymorphism or mechanism underlying their effect. In ideal circumstances, the gene will be identified, and the causal sequence polymorphism and/or mechanism can be proven or strongly inferred. Figure 2 shows a flowchart depicting the logical flow and depth of understanding that can be achieved for the gene and/or causal variant underlying a QTL or trait of interest.

There is a natural curiosity that drives the pursuit of causal variants, particularly those underlying domestication traits, classical morphological mutants, and traits of agricultural and economic importance. Furthermore, identifying the causal variants can yield new insight into the species of interest and unlock new opportunities for breeding and crop improvement communities, such as enhancing selection for favorable variants, screening resequencing datasets to identify accessions with potentially useful alleles, and developing novel variants through modern biotechnological approaches.

In this review, we examine genes that have been identified or validated in soybean (*Glycine max* (L.) Merr.) that underlie qualitative traits or QTLs that were previously mapped. We focus largely on the natural variants and exclude genes that were identified using induced variants. We also discuss the challenges associated with gene identification and the ways in which current tools/technologies are propelling the research community to identify and discover causal variants for previously recalcitrant QTL.

## 2 UTILIZING MODERN TOOLS FOR MAPPING AND GENE DISCOVERY AND IDENTIFICATION

Modern tools to pursue mapping and gene identification projects have advanced significantly in recent years, benefiting research in both model and non‐model systems. For many crop species, extensive genome sequence resources are now available, including near‐gapless reference genome assemblies and deep resequencing panels of numerous accessions. In soybean specifically, there are three new near‐gapless reference genome assemblies of Williams 82 (Espina et al., 2024; Garg et al., 2023; L. Wang et al., 2023) and a comprehensive pangenome of both cultivated and wild soybean [*Glycine soja* (Siebold & Zucc.)] (Y. Liu et al., 2020), which has proven useful for mining variation—as demonstrated by the identification of the *Rps11* locus (W. Wang, Chen, Fengler, et al., 2021). Moreover, the soybean research community has generated deep resequencing data for diverse germplasm panels (S. Liu et al., 2020; Valliyodan et al., 2021, 2016; Z. Zhu et al., 2025), which can be leveraged for high‐resolution mapping. The high density of variants in these datasets enhances the precision in pinpointing the potential causal polymorphisms.

**Core Ideas**

- Specific circumstances determine the likelihood of identifying the causal molecular variant underlying a phenotype.
- Advances in omics technologies have aided the identification of complex causal variants.
- Variants underlying crop domestication and agricultural traits may be more likely to be caused by complex variants.

Several mapping populations are publicly available to the soybean community, including classic isolines (Bernard et al., 1991), which have been widely used in many early gene identification studies (Gilbert et al., 2023). While some traits in these populations have already been coarsely mapped, there are still some traits for which the causal gene has not yet been identified (Gilbert et al., 2023). The soybean nested association mapping (SoyNAM) population (Diers et al., 2018; Q. Song et al., 2017) is another valuable resource that can be tapped for gene discovery, fine‐mapping, and potentially gene identification projects. Importantly, the cost of genotyping for mapping and fine‐mapping experiments has become increasingly affordable, making these approaches accessible to individual research programs.

Public mutant resources are available in soybean and may be useful for certain gene validation experiments. These include large mutant populations spanning different maturity groups (MGs) from MG I to MG VII (Stupar et al., 2024). These populations also were developed using different approaches giving the community different kinds of variants to explore, such as fast neutron populations where large structural deletions can be expected (Bolon et al., 2014; Prenger et al., 2019; Stacey et al., 2016) and some chemically mutagenized populations carrying single nucleotide polymorphism (SNP) mutations throughout the genome (Espina et al., 2018; Lakhssassi et al., 2021; Thapa et al., 2019). These mutant populations are useful for studying phenotypic mutations and can also be utilized to mine genotypic mutations to validate candidate genes using the Targeting Induced Local Lesions in Genomes (TILLING) approach. Few of the public mutant lines have been resequenced to date; however, future resequencing efforts would be an important step toward enabling reverse genetic approaches to functional genomics, allele mining, and candidate gene validation.

Another essential resource for fine mapping, gene validation, and causal variant identification is the suite of computational resources and databases that can be used to prioritize candidate genes. One such resource is the Soybean Expression Atlas (Almeida‐Silva et al., 2023), available through https://soyatlas.venanciogroup.uenf.br/. Additionally, the Plant Epigenome Database, which includes data for soybean (Z. Lu et al., 2019), provides valuable information on chromatin accessibility and histone modifications. These epigenetic features play a critical role in regulating gene expression and can help identify causal polymorphisms such as epiallele variants. Moreover, SoyBase also houses several other databases, such as soybean gene ontology, soybean metabolic pathways (SoyCyc), and pangenes from other legume species (Stupar et al., 2024). These resources can further support the identification of candidate genes that are functionally related. Furthermore, SoyKB (Joshi et al., 2014, 2012, 2017) is another powerful tool available for mining large‐scale omics data in the soybean community. Perhaps one of SoyKB's most important features is the Allele Catalog Tool (Chan et al., 2023), which displays alleles from different large‐scale resequencing panels to facilitate allele mining and finding potential causal variants associated with identified QTL.

Validation of the candidate gene is a critical step in the process of gene identification. In recent years, advances in biotechnology have significantly enhanced our ability to functionally validate candidate genes. The accessibility of genome engineering technology such as Clustered Regularly Interspaced Short Palindromic Repeats (CRISPR)‐CRISPR‐associated protein 9 (Cas9) has revolutionized gene discovery due to its high target specificity and efficiency (H. Zhu et al., 2020). In soybean, genome editing has been applied in numerous contexts (J. Liu et al., 2019), including gene validation. Many of the recently discovered genes have been validated using CRISPR‐Cas9, which enables precise knockout of candidate genes, facilitating assessment of the gene function.

The soybean research community and technological infrastructure are no longer limiting for many gene discovery projects. Indeed, these technologies have propelled the research community into discovering and identifying numerous genes as highlighted in the recent literature (Gilbert et al., 2023; Lemay et al., 2023; Tian et al., 2025) and summarized in Table 1.

**TABLE 1:** A list of soybean genes/quantitative trait locus (QTL) identified/validated with an inferred causal variant. The **√** symbol indicates the category assigned to the causal variant. The list includes only natural variant alleles (e.g., not alleles induced by random or targeted mutagenesis).

Trait	Locus	Gene model	Causal variant	Year	SNP/indel	Regulatory	TE	PAV/CNV	Epiallele	References
Shattering	*GmNST1A*	*Glyma.07G050600*	A > T in exon 3 resulting in premature stop codon	2025	**√**					(Z. Zhu et al., 2025)
Seed oil‐to‐protein ratio	*GmSOP20*	*Glyma.20G005900*	C/CACT in exon 2 and CAAC/C in exon 3	2025	**√**					(Zheng et al., 2025)
Leaflet number	*Lf2*	*Glyma.11G027100*	2‐bp deletion in exon 4	2025	**√**					(Clark et al., 2025)
Resistance to Asian soybean rust	*Rpp6907*	A 60 kb region between the genes *Glyma.18G283100* and *Glyma.18G283300* in the SX6907 genome	57 kb CNV	2024				**√**		(Hao et al., 2024)
Shattering	*sh1*	*Glyma.16G141100*	9‐bp deletion, A > G mutation	2024	**√**					(S. Li et al., 2024)
Growth habit, pubescence form, main stem length, leaf size, stem twining, and leaf hopper resistance	*qGH‐12, qPB‐12, qMSL‐12, qLSZ‐12, and qST‐12*	*Glyma.12G213800* and *Glyma.12G213900*	CpG methylation in promoter region of *lncRG1* and *lncRG2*	2024					**√**	(W. Wang et al., 2024)
Drought tolerance	*GmPrx16*	*Glyma.16G16440* *0*	C > G in exon 1	2024	**√**					(Z. Zhang et al., 2024)
Male sterility	*ms2*	*Glyma.10G281800*	17‐bp deletion and 16‐bp insertion in exon 3	2023	**√**					(X. Fang et al., 2023; Yu et al., 2024)
Pubescence shape	*Mao1*	*SoyC08_13G189600*	TE insertion in the promoter	2023			**√**			(An et al., 2023)
Shattering and pod pigmentation	*L1*	*Glyma.19G120400*	Arginine to Cysteine (R31C)	2023	**√**					(Lyu et al., 2023)
Internode length	*PH13*	*Glyma.13G276700*	*Ty1*/*copia*‐like retrotransposon insertion	2023			**√**			(Qin et al., 2023)
Resistance to soybean cyst nematode	*GmSNAP02*	*Glyma.02G260400*	22‐bp deletion in exon1	2023	**√**		**√**			(Usovsky et al., 2023)
Seed size and quality	*GmST05*	*SoyZH13_05G229200*	Indels in the promoter region	2022		**√**				(Duan et al., 2022)
Seed protein	*cqSeed protein‐003/* *POWR1*	*Glyma.20g085100*	321‐bp deletion in exon 4	2022	**√**					(Fliege et al., 2022; Goettel et al., 2022)
Flowering time and growth habit	*Tof18/SOC1*	*Glyma.18G224500*	G > A in the promoter	2022		**√**				(Kou et al., 2022)
Resistance to *soybean mosaic virus*	*GsRSS3L*	*Glyma.17g238900*	Ser > Phe in exon 1 and Cys > Thr in exon 3	2022	**√**					(S. Song et al., 2022)
Flowering time	*QNE1*	*Glyma.06G204300*	C686G and C1063T in coding region	2022	**√**					(Xia et al., 2022)
Male sterility	*ms3*	*Glyma.02G107600*	W628V, premature stop codon	2022	**√**					(Hou et al., 2022)
Male sterility	*ms1*	*Glyma.13G114200*	Deletion in *Glyma.13G114200*	2021				**√**		(X. Fang et al., 2021; B. Jiang et al., 2021; Nadeem et al., 2021)
Resistance to *Phytophthora sojae*	*Rps11*	27.7 kb NBS‐LRR gene in the PI 594527 genome	27.7 kb CNV	2021				**√**		(W. Wang, Chen, Fengler, et al., 2021)
Plant Height	*S*	*Glyma.13G287600* and *Glyma.13G288000*	74 kb CNV	2021				**√**		(X. Wang, Li, et al., 2021)
Male sterility	*ms6*	*Glyma.13G066600*	T330A in exon 2 resulting in L76H	2021	**√**					(Yu et al., 2021)
Seed size	*qSw17‐1/GmKIX8‐1*	*Glyma.17G112800*	Promoter variation	2021		**√**				(Nguyen et al., 2021)
Flowering time	*Tof12/GmPRR3b*	*Glyma.12G073900*	C1879T leading to premature stop codon	2020	**√**					(C. Li et al., 2020; S. Lu et al., 2020)
Flowering time	*Tof11/GmPRR3a*	*Glyma.11G148362*	A2210‐ leading to frameshift	2020	**√**					(S. Lu et al., 2020)
Pubescence density	*Pd1*	*Glyma.01G240100*	T > C in last exon (V‐A in C terminus)	2020	**√**					(Liu et al., 2020)
Pubescence density	*P1*	*Glyma.09G278000*	A > G in exon 1 (A25T)	2020	**√**					(Liu et al., 2020)
Pubescence density	*Ps*	*Glyma.12G187200*	25.6 kb CNV	2020				**√**		(Liu et al., 2020)
Pubescence color	*Td*	*Glyma.03G258700*	Premature stop codon	2019	**√**					(Yan et al., 2019)
Male sterility	*ms4*	*Glyma.02G243200*	Frameshift mutation in exon 3 leading to premature stop codon	2019	**√**					(Thu et al., 2019)
Resistance to soybean cyst nematode; interacts with Rhg1	*QTN07/NSF_RAN07_*	*Glyma.07G195900*	C‐terminal amino acid polymorphism	2018	**√**					(Bayless et al., 2018)
Seed coat color	*G*	*Glyma.01G198500*	SNP caused splice variant in exon 9	2018	**√**					(M. Wang et al., 2018)
Seed coat bloom	*B1*	*Glyma.13G241700*	T > C in exon 2	2018	**√**					(D. Zhang et al., 2018)
Flowering time	*J*	*Glyma.04G050200*	10‐bp deletion, missense, and premature stop codon	2017	**√**					(S. Lu, Zhao, et al., 2017)
Seed size	*PP2C‐1*	*Glyma.17G221100*	Amino acid substitutions in N‐terminus	2017	**√**					(X. Lu, Xiong, et al., 2017)
Pubescence shape	*p2*	*Glyma.20G019300*	1‐bp deletion leading to premature stop codon	2016	**√**					(Campbell et al., 2016)
Hardseededness	GmHs1‐1	*Glyma.02G269500*	C > T mutation in exon 8	2015	**√**					(Sun et al., 2015)
Foliage pigmentation	*Y11 and CD‐5*	*Glyma.13G232500* and *Glyma.15G080200*	A > G and G > A in exon 3	2014	**√**					(Campbell et al., 2014)
Shattering	*Pdh1*	*Glyma.16G141400*	Premature stop codon	2014	**√**					(Funatsuki et al., 2014; S. Li et al., 2024)
Shattering	*SHAT1‐5*	*Glyma.16G019400*	20‐bp deletion in promoter region	2014		**√**				(Dong et al., 2014)
Chlorophyll retention	*d1*	*Glyma.01G214600*	1‐bp deletion in exon 2	2014	**√**					(C. Fang et al., 2014)
Chlorophyll retention	*d2*	*Glyma.11G027400*	GmD2IN insertion in exon 4	2014			**√**			(C. Fang et al., 2014)
Salt tolerance	*GmSalt3*	*Glyma.03G171600*	3.78 kb *copia* transposon insertion leading to premature stop codon	2014			**√**			(Guan et al., 2014)
Resistance to soybean cyst nematode	*Rhg1*	31 kb segment at rhg1‐b locus	31 kb CNV and indels in the repeat junction	2012	**√**			**√**		(Cook et al., 2012)
Resistance to soybean cyst nematode	*Rhg4*	*Glyma.08g108900*	R130P in exon 1 and Y358N in exon 3	2012	**√**			**√**		(S. Liu et al., 2012; Patil et al., 2019)
Leaf shape and number of seeds per pod	*Ln*	*Glyma.20G116200*	Asp (Ln) to His (ln) in exon 2	2012	**√**					(C. Fang et al., 2013; Jeong et al., 2012)
Flowering time	*E1*	*Glyma.06G207800*	1‐bp deletion, TE insertion, and 1 allele deleted out	2012	**√**		**√**	**√**		(Xia et al., 2012)
Flowering time	*e2*	*Glyma.10G221500*	Premature stop codon	2011	**√**					(Watanabe et al., 2011)
Hilum color and seed coat color	*R*	*Glyma.09G235100*	Nucleotide substitution in exon 2, frameshift mutation	2011	**√**					(Gillman et al., 2011)
Growth habit	*dt1*	*Glyma.19G194300*	Four nucleotide substitution in *GmTfl1*	2010	**√**					(Tian et al., 2010)
Flower color	*w4*	*Glyma.17G252200*	20.5 kb TE insertion in second intron	2010			**√**			(M. Xu et al., 2010)
Flowering time	*e3*	*Glyma.19G224200*	Deletion in exon 3	2009	**√**					(Watanabe et al., 2009)
Flowering time	*E4*	*Glyma.20G090000*	*Ty1*/*copia*‐like retrotransposon insertion in exon 1	2008			**√**			(B. Liu et al., 2008)
Flower color	*Wm*	*Glyma.13G082300*	1‐bp deletion in coding region	2007	**√**					(Takahashi et al., 2007)
Flower color	*w1*	*Glyma.13G072100*	65‐bp insertion in exon 3	2007	**√**					(Zabala & Vodkin, 2007)
Pubescence color	*T*	*Glyma.06G202300*	1‐bp deletion cause frameshift	2002	**√**					(Toda et al., 2002; Zabala & Vodkin, 2003)
Seed coat color	*I*	Cluster of CHS genes on Gm08	10.9 kb inversion/duplication	1996				**√**		(Todd & Vodkin, 1996; Tuteja et al., 2009; Vodkin et al., 2025)

Abbreviations: CHS, chalcone synthase; CNV, copy number variation; indel, insertion/deletion; NBS‐LRR, Nucleotide‐binding site Leucine‐rich repeat; PAV, presence/absence variation; RNAi, RNA interference; SNP, single nucleotide polymorphism; TE, transposable element.

## 3 WHAT SEQUENCE VARIANTS UNDERLIE QTL?

**FIGURE 3:**
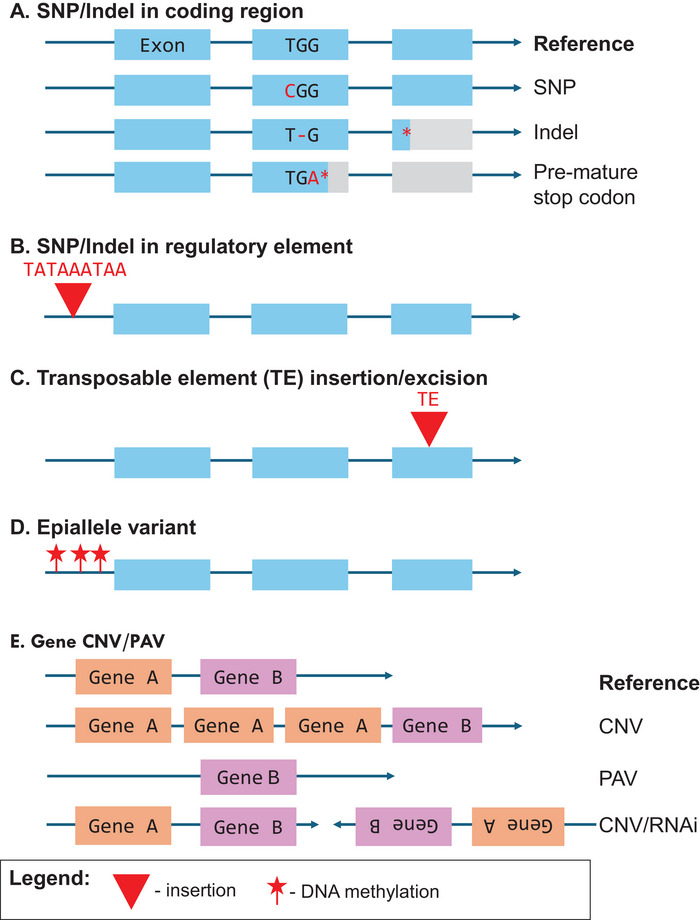
Types of sequence polymorphisms underlying a quantitative trait locus (QTL). (A) Single nucleotide polymorphism (SNP)/insertion/deletion (indel) in coding region: SNPs or insertions/deletions (indels) occurring within exons may alter the encoded amino acid, introduce premature stop codons, or result in frameshift mutations, potentially disrupting protein function. (B) SNP/indel in regulatory element: Variation in non‐coding regulatory regions, such as the insertion in the promoter (red triangle) can influence gene expression levels. SNP variants in the regulatory regions (not shown) can also influence expression states. (C) Transposable element (TE) insertion/excision: Insertion of a TE (red triangle) within or near a gene can disrupt gene structure. (D) Epiallele variant: Epigenetic modifications, such as differential DNA methylation (red stars), can alter gene regulation and phenotype. Excision of TE elements (not shown) can also alter gene structure and function. (E) Gene CNV/PAV: Structural variations such as copy number variation (CNV) or presence/absence variation (PAV) lead to changes in gene dosage or regulation. Not shown: Large chromosomal translocations. RNAi, RNA interference.

The plethora of recent discoveries provides the necessary background information to ask a simple but surprisingly difficult question: What are the sequence variants that underlie QTL? Figure 3 illustrates the different types of sequence polymorphisms that may underlie a QTL, ranging from SNPs to large and complex structural variants. In this section, we examine the existing literature on the identified causal genes in soybean, highlighting some examples of different sequence polymorphisms and how they impact gene function (Table 1). We also pose the question, what is the frequency of each variant class in determining trait/QTL variation? Are there specific types of variants that are more frequently identified as causal compared to other types of variants? By mining the literature, we aim to uncover trends in the types of sequence variation most often implicated in trait variation.

### 3.1 SNP/insertion/deletion in coding regions

In the modern literature, there are examples of several different variant types, such as SNP or small insertion/deletion (indel) polymorphisms that disrupt gene coding regions or exons (Figure 3A). SNPs within coding regions can result in nonsynonymous mutations that alter amino acid sequences, affecting protein structure or function and ultimately influencing the phenotype (G. R. Kumar et al., 2010). For example, in soybean, a G‐to‐A nonsynonymous SNP in the first exon of the lipid transfer protein gene (gene model *Glyma.09G278000*) leads to an amino acid substitution from Arg25Thr, which leads to glabrous pubescence at the *P1* locus (Liu et al., 2020). An SNP in the coding region can also introduce a premature stop codon, truncating the protein sequence and disrupting the function. A well‐characterized case is the *e2* locus controlling flowering time in soybean, where an SNP in the 10th exon of *GmGIa* (*Glyma.10G221500)* resulted in a premature stop codon and is associated with early flowering (Watanabe et al., 2011). Similarly, a small indel in the coding region can also impact gene function, particularly when it introduces a frameshift or deletes an important conserved domain, thereby affecting the functionality of the protein sequence. A notable example of this case is the soybean *J* locus, also involved in flowering time regulation, where a natural variant in the BR121 soybean line harbored a 10‐bp deletion causing a frameshift and resulting in early protein termination after 195 amino acids for the gene model *Glyma04G050200*. The truncation of this protein (normally 714 amino acids in length) removes its function, leading to a phenotype that prolongs soybean maturity and enhances the adaptation of soybean in low‐latitude regions (S. Lu, Zhao, et al., 2017).

Based on the literature, SNP/indels within coding regions are among the most frequently observed causal variants underlying a QTL. Table 1 provides a categorization of causal variants for 62 different alleles previously published in soybean. The majority of these alleles (40 out of the 62) have been attributed to SNPs/indels within coding regions. However, one may argue that SNPs and indels are relatively easier to find compared to other variant types, as current tools such as resequencing and allele mining databases readily identify such variants. Thus, it is not clear whether SNPs/indels truly underlie a majority of trait/QTL variants, or if this is an overestimation based on a systematic discovery bias.

### 3.2 SNP/indel in regulatory regions

Sequence variants in non‐coding regions, particularly within regulatory elements such as promoters, untranslated regions, enhancers, and introns, can also significantly impact gene function (e.g., Figure 3B). Indeed, meta‐analysis of human GWAS datasets indicates that potential causal variants are enriched in both gene coding regions and regulatory elements (Watanabe et al., 2019). Regulatory variants can influence transcript synthesis and accumulation, thereby affecting the expression levels of a gene (G. R. Kumar et al., 2010). For instance, a 20‐bp deletion in the promoter region of *Glyma.16G019400*, which encodes for *SHATTERING1‐5 (GmSHAT1‐5)*, was identified in the cultivated soybean (Dong et al., 2014). This deletion disrupted a core binding site, resulting in a 15‐fold increase in expression of *GmSHAT1‐5* compared to the wild soybean *G. soja*, where this change in expression is associated with pod shattering resistance (a very important domestication trait difference between wild and cultivated soybean) (Dong et al., 2014). Compared to SNPs/indels within coding regions, sequence polymorphisms in the regulatory regions are oftentimes not as straightforward to identify and assess causality/effects on gene function, but their effects can be just as important. Understanding how regulatory elements influence and regulate traits of interest can be informative for developing/modifying subtle changes in phenotype. A classic example is promoter editing performed in tomato (*Solanum lycopersicum* L.), wherein targeting specific regulatory elements provided a spectrum of quantitative variation in fruit size, plant architecture, and inflorescence branching (Rodríguez‐Leal et al., 2017). In‐depth characterization of soybean *cis*‐regulatory elements (X. Zhang et al., 2025) will be useful for advancing this area of research in soybean.

### 3.3 Transposable element insertion/excision

Transposable elements (TEs) are abundant in plant genomes, and the movement of these elements, such as insertions and excision, contributes significantly to genetic variation and in shaping the plant genome (Hirsch & Springer, 2017). Although not as abundant as in maize (*Zea mays* L.), the soybean genome is composed of approximately 60% TEs, including DNA transposons, *copia‐*, and *gypsy*‐like retrotransposons (Oliver et al., 2013; Schmutz et al., 2010). The insertion/excision of TEs can have several consequences that lead to phenotypic changes, such as disruption of a coding region resulting in an altered or truncated coding sequence (Figure 3C), generation of alternative splicing events, and creation of alternative start and stop codons (Della Coletta et al., 2021). Moreover, TEs can influence gene expression through various mechanisms, such as impacts on promoter function and disruption of *cis*‐regulatory sequences (Hirsch & Springer, 2017).

There are some published accounts in soybean wherein TE variation was inferred to underlie QTL variation. A prime example is the photosensitivity locus (*E4*), where a recessive allele of *GmphyA2* (gene model *Glyma.20G090000*) contains a *Ty1/copia*‐like retrotransposon insertion in exon 1, leading to insensitivity to long‐day photoperiod (B. Liu et al., 2008). Meanwhile, variation at the *Mao1* locus, which affects pubescence form, is driven by a *Ty3/gypsy* retrotransposon insertion in the promoter region, approximately 1.7 kb upstream of the initiation codon of *Mao1* (*SoyC08_13G189600*). This TE insertion results in upregulation of the *Mao1* gene and an erect pubescence form (An et al., 2023).

TEs can influence and affect traits through a wide range of mechanisms, including chromatin alterations. Therefore, identifying TE disruption as the causal polymorphism underlying a QTL or candidate gene can be challenging, particularly when the responsible TE variant is located outside of a gene reading frame.

### 3.4 Epiallele variant

Epigenetic alleles (epialleles) are heritable allele variants that underlie phenotypic variation but are not caused by DNA sequence per se. Instead, an epiallele may be driven by variation in chromatin state (Hofmeister et al., 2017). Some epialleles may be caused by changes in DNA methylation patterns (Figure 3D) or post‐translational histone modifications (Agarwal et al., 2020). Epiallele changes often influence the phenotype by affecting gene expression levels (Springer, 2013). In soybean, the first known epiallele implicated in a QTL was recently identified, involving two tandem *long non‐coding RNA genes* (*lncRG*), *Glyma.12G213800* and *Glyma.12G213900*, which underlie multiple domestication traits, including pubescence form, main stem length, leaf size, growth habit and stem twining, and leafhopper resistance (W. Wang et al., 2024). In this study, *lncRG1 and lncRG2* were found to be highly similar to the MYB transcription factor, suggesting that they were derived from MYB genes, with each lncRNA gene containing inverted repeats of MYB coding sequence (W. Wang et al., 2024). The authors observed an increase in CpG methylation in the promoter region of *lncRG1 and lncRG2* associated with reduced expression levels in the cultivated soybean lines, leading to altered regulation of the downstream MYB genes and observed changes in the domestication traits (W. Wang et al., 2024).

Identifying an epiallele as a causal polymorphism for a candidate gene or QTL is particularly challenging, as they require different assay types (e.g., bisulfite sequencing) than are typically used in the course of mapping a locus. Assessing loci may be complicated, as an epiallele may be linked to a SNP or other DNA marker variant, which may lead the researcher to misidentify the DNA variant as the causal feature. Furthermore, identification could be affected by the age and stability of the epigenetic modification (Noshay & Springer, 2021). Understanding that gene expression provides a primary conduit for epigenetic variation to affect phenotype is critical (Springer, 2013), as shown in the case study above. Increased availability of comparative epigenomic datasets (e.g., methylome sequencing, chromatin accessibility, and histone modifications) of soybean will provide resources to screen for epiallele variants within QTL regions of interest. Examples of in‐depth soybean epigenomic profiles (e.g., C. Zhang et al., 2023; X. Zhang et al., 2025) and some comparative studies (e.g., Kim et al., 2015; Schmitz et al., 2013; Shen et al., 2018) have been published.

### 3.5 Gene copy number variation (CNV)/presence/absence variation (PAV) and other structural rearrangements

CNV and PAV are structural rearrangements of DNA segments involving gains or losses of genomic material (Figure 3E), typically larger than 1 kb (Żmieńko et al., 2014). CNVs and PAVs can contribute to phenotypic variation through several mechanisms, oftentimes through the deletion of protein‐coding genes, leading to protein loss of function. These structural variants can also have dosage effects, where an increased number of gene copies may influence gene expression levels (Lye & Purugganan, 2019). CNVs/PAVs have been implicated as the causal polymorphism in many disease resistance genes, particularly in nucleotide‐binding leucine‐rich repeat gene clusters, which frequently exhibit CNV across different cultivars and species (Dolatabadian et al., 2017). It is hypothesized that this results, in part, from the gene‐for‐gene resistance mechanism, where rapid recognition specificity in defense responses drives frequent expansion and contraction of disease‐resistance gene families (Dolatabadian et al., 2017). In soybean, the discovery of soybean cyst nematode (SCN) resistance locus *Rhg1* is a well‐known case study of a copy number variant implicated as causal polymorphism. In this case, a 31‐kb multi‐gene cluster was observed to have increased copy number in several SCN‐resistant soybean accessions (Cook et al., 2012). A notable case in soybean where the absence of a gene model resulted in a phenotypic variation is the *E1* locus. In this example, one of the alleles, *e1‐nl*, has a 130‐kb deletion that completely removes the entire *E1* gene (*Glyma.06G207800*), leading to photoperiod insensitivity where cultivars carrying this allele exhibit early flowering in high latitude area (Xia et al., 2012).

In addition to deletions and duplications, structural variation also includes chromosomal translocations and inversions, which can influence trait variation and species boundaries. For example, a translocation between chromosomes 11 and 13 has been identified as the cause of semi‐fertility in hybrids between cultivated and wild soybean (W. Wang, Chen, Wang, et al., 2021). Furthermore, induced structural variants have also been used to identify gene functions in soybean. For example, an induced translocation led to the identification of a gene involved in seed sucrose and oil content (Dobbels et al., 2017).

Perhaps the most complex natural structural variant underlying a QTL in soybean is the *I* locus, which controls seed coat pigmentation patterning (Todd & Vodkin, 1996; Tuteja et al., 2009). Alleles at this locus produce small interfering RNAs (siRNAs) that downregulate chalcone synthase (CHS) mRNA targets. The siRNAs are differentially expressed among the different *I* locus alleles, in turn leading to the differential expression of the CHS genes that cause the differential seed coat pigmentations. While the mechanism of silencing is post‐transcriptional, it appears that the cause of the differential siRNA production is genetic, based on a series of large duplications, inversions, and deletions among the *I* locus alleles (see the “CNV/RNAi” diagram in Figure 3E as an example of such rearrangements). The *I* locus structural variants cause differential production of siRNAs that silence CHS mRNAs, leading to a complex range of different seed coat pigmentation phenotypes (Vodkin et al., 2025). Therefore, Table 1 classifies the *I* locus as a PAV/CNV causal variant rather than an epiallele or some other category.

## 4 WHAT MAKES A CAUSAL VARIANT DIFFICULT TO IDENTIFY?

Although a large number of traits have been mapped and many genes have been identified and validated, there are still numerous traits for which the causal genes have not been identified (Gilbert et al., 2023) or where the genes have been identified but the causal polymorphisms have yet to be inferred, as shown in Table 2. Several factors influence the likelihood of a causal variant to be discovered. In this section, we will address some of these key factors and highlight why some genes were discovered and characterized relatively quickly, while other genes took decades before being implicated and their causal variants were inferred. This section builds upon a vast amount of scientific literature and previous perspectives articles that explore the relative merits of mapping strategies (e.g., biparental vs. association mapping), gene validation platforms, and the adoption of technologies to facilitate the identification of causal variants (e.g., Colasuonno et al., 2021; Jaganathan et al., 2020; Kaňovská et al., 2024; Liang et al., 2021; C. Miao et al., 2019).

**TABLE 2:** Examples of soybean genes identified/validated without inference of the causal variant.

Trait	Locus	Gene	References
Resistance to soybean cyst nematode	*qSCN10*	*Glyma.10g194800* and *Glyma.10G196400*	(Lakhssassi et al., 2025)
Resistance to soybean cyst nematode	*Rscn‐16/GmUGT88A1*	*Glyma.16G175200*	(H. Jiang et al., 2025)
Resistance to *Phytophtora sojae*	*RpsYD29*	*Glyma.03g033600*	(W. Li et al., 2023)
Resistance to soybean cyst nematode	*cqSCN‐006*	*Glyma.15G191200*	(Butler et al., 2021)
Seed size, oil content, and protein content	*GmSWEET39/GmSWEET10a*	*Glyma.15G049200*	(L. Miao et al., 2020; S. Wang et al., 2020)
Growth habit	*Dt2*	*Glyma.18G273600*	(Ping et al., 2014)

### 4.1 Lack of clear and accessible phenotype

Identifying causal genes is easier when there is a clear, affordable, and accessible phenotype. Qualitative traits, such as flower color, are relatively straightforward, as they produce distinct and observable phenotypes compared to quantitative traits such as disease resistance genes, which are typically influenced by environment and multiple genes, making phenotyping more complex and resource intensive (Fu et al., 2023). In some cases, such as with abiotic stress traits, inducing a consistent phenotype in a controlled environment can be particularly challenging, making the molecular and physiological validation of any candidate gene difficult (Merry et al., 2022). The genetic architecture of a trait also influences the likelihood of its causal gene(s) being identified. Qualitative traits, typically controlled by a single gene, are generally easier to dissect, while quantitative traits are often controlled by multiple genes of small effect, requiring phenotyping of large populations for adequate power to detect and estimate their effects (T. F. Mackay, 2001; Remington et al., 2001). Genetic architecture is discussed in more detail below, in Section 4.5.

Phenotyping becomes even more complicated when a trait exhibits strong genotype‐by‐environment interactions, a common phenomenon among quantitative traits. In such cases, individual phenotype measurement from a single environment would not allow for a reliable estimate of the QTL effect (T. F. Mackay, 2001). Beyond biological complexity, phenotyping can also be limited by practical constraints, including cost, labor intensity, and the need for destructive sampling, all of which can affect functional genomics studies (Yang et al., 2020). In recent years, however, the development of better high‐throughput phenotyping technologies has helped to improve mapping and gene discovery projects. These technologies enable non‐destruction sampling, enhance phenotypic accuracy, enable greater replication, and facilitate large‐scale screening (Mir et al., 2019).

### 4.2 Mapped interval has no clear candidate gene

Map‐based cloning often narrows the genomic interval to a very small region containing a handful of candidate genes to be evaluated, but identifying the causal gene within the interval can still be challenging (Salvi & Tuberosa, 2005). Candidate gene identification is more straightforward when gene models within the mapped interval have been annotated or share homology or synteny with genes from well‐characterized model organisms, such as Arabidopsis (*Arabidopsis thaliana*), rice (*Oryza sativa* L.), or barrel medic (*Medicago truncatula* Gaertn.) (Salvi & Tuberosa, 2005). When a candidate gene exhibits such homology to a model organism, additional resources such as mutant stocks from the model organism can be leveraged for gene complementation and validation experiments. Traits associated with conserved biological processes or metabolic pathways across plant species are typically easier to dissect because information from other model species can help prioritize candidate genes (Bargsten et al., 2014). For instance, the identification of the soybean *Dt1* gene, controlling determinate growth habit, was informed by homology between their candidate gene and the previously characterized Arabidopsis *terminal flower 1* (*TFL1*), a gene involved in meristem development (Tian et al., 2010). Furthermore, the authors were able to use the Arabidopsis *tfl1* mutant to functionally validate the soybean candidate gene *GmTfl1* for determinate growth habit (Tian et al., 2010). In contrast, traits not shared with model systems, such as soybean SCN resistance, are often more difficult to identify due to a lack of homologous genes, functional annotations, and previous characterization in model species. Generally speaking, any knowledge about the gene models in a mapped interval can be highly valuable for accurate candidate gene identification.

### 4.3 Abundance of sequence polymorphism

When a mapped interval is sufficiently narrow, it may contain a small number of potential causal variations, such as SNPs, indels, and structural variants located in the coding and non‐coding regions. With the availability of high‐density markers and whole‐genome resequencing data, haplotype analysis can help determine the most likely causal variant, as is widely used in GWAS‐based approaches (Bhat et al., 2021). In many cases, variants such as SNPs and indels are associated with the trait of interest, particularly if they are predicted to affect gene coding regions and protein outputs. However, in some instances, no clear sequence polymorphisms are detected. Conversely, too much sequence polymorphism can also be problematic, as it may lead to the identification of more candidate genes than can be reasonably screened by validation approaches (Figure 1).

In some cases, complex variant types, such as long non‐coding RNAs, highly repetitive TEs, large CNVs, or epialleles, may underlie the trait. In some cases, alternative approaches to pinpoint the variants may be necessary, such as including bisulfite sequencing to detect epialleles. Furthermore, complex DNA sequence variants may be recalcitrant to accurate genome assembly and difficult to resolve using short‐read sequencing technology (Della Coletta et al., 2021). For example, a large CNV present in the mapping parent but absent from the reference genome may not be detected due to reference bias, potentially leading to missed association between the causal variant and the phenotype (Della Coletta et al., 2021). In such cases, the use of pangenomes and near‐gapless assemblies may better capture the true genomic diversity and help overcome these limitations. For example, W. Wang, Chen, Fengler et al. (2021) used a soybean pangenome (Liu et al., 2020) as a reference for comparison with a landrace carrying *Rps11*. They were able to identify a unique R‐gene in the landrace (and not found in the pangenome) conferring resistance to at least 12 *Phytophthora sojae* races.

### 4.4 Location in the genome

The resolution of mapping and fine‐mapping a locus is largely determined by the location of the QTL in the genome. In some cases, initial mapping may be accurate enough to bypass the fine‐mapping process (Price, 2006), whereas other loci may be recalcitrant to high‐resolution mapping. The ability to create a high number of recombinants within a relatively small interval is beneficial for fine‐mapping, and this is more feasible in many species if the location of the QTL is toward the end of the chromosomes, where recombination rates are typically higher (Remington et al., 2001). In contrast, QTLs that map to regions where the recombination rate is lower, such as centromeric or pericentromeric regions, are more difficult to resolve. In some cases, it can take decades before the genes can be fully characterized and validated (Fliege et al., 2022; Remington et al., 2001). Structural variations can further influence the recombination rate. For instance, TEs tend to accumulate in regions with low recombination and in turn can suppress recombination (Stapley et al., 2017). In soybean, around 20% of genes are in the pericentromeric region, which only accounts for 7% of recombination (Du et al., 2012; Schmutz et al., 2010). The relative timing of a causal mutation event versus the mutations creating markers, alongside historical recombination within linked regions, can also influence detection power and mapping resolution. For example, if a causal mutation occurred relatively recently and/or is infrequently represented in the germplasm, it may not be detected by association mapping approaches. Furthermore, gene validation can be influenced by genome localization as well. If genetic transformation is used, the location of gene insertions will affect the expression of the transgene due to position effects based on local chromatin structure. Areas of higher nucleosome density, such as heterochromatic regions, have lower transformation efficiency and may be more apt to silence transgenes (Aslankoohi et al., 2012).

### 4.5 Genetic architecture

The genetic architecture of a trait strongly influences the ability to accurately and precisely map QTLs that explain its variation, which in turn influences the probability of identifying causal variants. QTLs of major effect are obviously the most likely to be identified. For quantitative traits under polygenic genetic control, identifying causal variants is oftentimes challenging due to their intricate genetic architecture. For example, some traits may be controlled by a large number of genes, each with a small effect. As the genetic control of a trait approaches the infinitesimal model (Barton et al., 2017), the effects of individual loci would be too small to detect, making QTL mapping, gene validation, and causal variant detection extremely difficult for such traits. The “omnigenic model” is a variation of the infinitesimal model that attempts to add a mechanistic basis explaining why the infinitesimal model works so well in practice (Boyle et al., 2017). Under the omnigenic model, a set of “core genes” involved in biochemical pathways directly related to the trait exist, but these core genes are influenced by a much larger set of “peripheral genes” distributed across the genome that interact with the core genes through regulatory networks. Because the peripheral genes are much larger in number than the core genes, they explain much of the heritability despite having very small effects. In addition to postulating a mechanistic basis to the infinitesimal model, the omnigenic model also provides hypotheses for the biological mechanisms underlying pervasive epistasis, manifesting itself into the so‐called genetic background effects that can alter QTL effects across divergent populations (T. F. C. Mackay, 2014). Likewise, pervasive epistasis can also severely hinder gene validation efforts. A given QTL, perhaps containing a core gene as the causal variant, may be detectable in one genotype due to certain combinations of alleles of peripheral genes in the genetic background. Identification of causal variants may fail, however, if the targeted mutations are created in a genetic background not harboring the interacting combinations of alleles found in the original genotype where the QTL was mapped. Good examples of epistatic interactions have been described in soybean SCN resistance, where resistance is dependent on the interactions of different SCN allelic combinations (Patil et al., 2019; Usovsky et al., 2023). In one example, varying copy number combinations of Rhg1 and Rhg4 loci affect the resistance response to different SCN races (Patil et al., 2019).

### 4.6 Difficult validation

Gene validation is a valuable but resource‐intensive endeavor, and its effectiveness depends on the quality of the validation approach. There is generally a trade‐off between efficiency and accuracy wherein more complex and effective validation methods may be time‐consuming and associated with greater costs (Figure 1). In regions with several candidate genes, a researcher may opt for a faster method in order to assess a greater number of candidates.

In some cases, ideal validation approaches may not be accessible due to technical limitations. For instance, developing a gene‐edited knockout allele may not be feasible if the species or genotype carrying the allele of interest is recalcitrant to transformation (though the development of genotype‐independent transformation methods is an active area of research for crop species; Maren et al. [2022]). Genetic background can affect validation outcomes in numerous other ways as well. For example, a gene that is knocked out or silenced in a background that already harbors a weak allele may not produce an observable phenotypic change. In other situations, a gene knockout or overexpression may generate unexpected pleiotropic effects. In extreme cases, this may include severely altered plant development or lethality. In less severe cases, pleiotropic effects may alter or mask the phenotype of primary interest, complicating the interpretation of the results.

Additionally, challenges in phenotyping and the need to evaluate plants under specific field conditions may impede progress in candidate gene validation. This may be particularly restrictive for transgenic and gene‐edited plants, which may be subject to regulatory permits that constrain the field environments in which they can be evaluated.

## 5 FUTURE PERSPECTIVES FOR GENE IDENTIFICATION AND DISCOVERY IN CROPS

In crop plant species, numerous genes underlying qualitative traits and QTL have been successfully identified. However, many candidate genes remain to be validated, and many causal polymorphisms remain to be discovered. While this article highlights the state of causal variant discovery in soybean, the ideas and themes presented in the sections above and here are applicable to any crop species.

**FIGURE 4:**
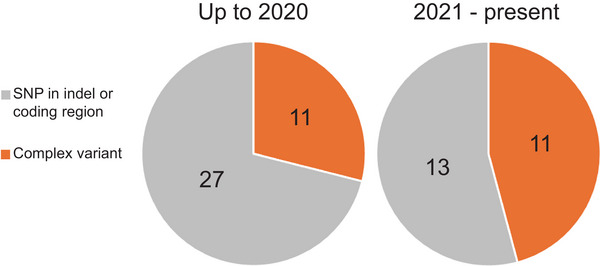
The distribution of causal variant types for cloned soybean genes over different timeframes. The number of causal variants per category per timeframe are shown within the pie charts. “Complex variant” categories include single nucleotide polymorphism (SNP)/insertion/deletion (indels) in regulatory elements, transposable element insertion/excision, epiallele variants, and gene content variants (copy number variation/presence/absence variation). Note the relative increase in the discovery rate of complex variants in more recent years.

This review highlights that most identified causal genes fall into one of three categories (see Table 1): (1) genes underlying historical morphological traits, (2) genes with functionally characterized homologs in model systems, and (3) more rarely, genes underlying agronomically or economically important traits. The latter group is typically more difficult to discover since the underlying genetics tend to be more quantitative and complex. Examination of causal polymorphisms underlying soybean traits found an enrichment of SNPs and indels within coding regions (Table 1). In contrast, more complex variants were identified less frequently. As discussed above, it is not clear if this pattern is caused by a true biological trend, or a discovery bias favoring polymorphisms that are more readily discovered. Furthermore, this may feed misconceptions about causal variants and the language scientists use to describe them. For example, the commonly used term quantitative trait nucleotide implies that a variant underlying a QTL should track to a single nucleotide. As discussed in this perspective, that is sometimes but not always the case. Indeed, there is evidence that the discovery of causal variants in soybean in recent years has shifted from predominantly SNPs/indels in coding regions to a suite of more complex variants (Figure 4). It would be interesting to examine if this trend is also observed in other crop species.

We speculate that technological advancements (e.g., better multi‐omics approaches) are rapidly improving our capacity for gene discovery and detection of more complex causal variants in crop species. We suspect this trend will continue, for at least two reasons. First, as mentioned above, improved omics methodologies have improved the scale and resolution of mapping and molecular dissection of QTL, increasing the likelihood that more complex variants will be identified. Second, as these capacities improve, researchers may be more likely to identify and validate causal genes that underlie QTL of agricultural importance, such as biotic and abiotic stress resistance genes that may be more likely to evolve by complex molecular mechanisms. These factors, coupled with increased technical capacity (e.g., pangenomes, enhanced machine learning, and an expanded biotechnology toolbox), will increase the feasibility of complex gene/variant discovery and applying these findings toward crop breeding and improvement.

## AUTHOR CONTRIBUTIONS

**Mary Jane C. Espina**: Conceptualization; visualization; writing—original draft; writing—review and editing. **Aaron J. Lorenz**: Conceptualization; supervision; writing—review and editing. **Robert M. Stupar**: Conceptualization; supervision; visualization; writing—original draft; writing—review and editing.

## CONFLICT OF INTEREST STATEMENT

The authors declare no conflicts of interest.

## Data Availability

Data summarized and associated with this article are provided in the figures and tables. Referenced data are available in the literature.
